# Comparison of Measurement Techniques for Photoreceptor Loss in Geographic Atrophy

**DOI:** 10.1167/tvst.15.7.8

**Published:** 2026-07-08

**Authors:** Maria Schulz, Nathan Macha, Sairi Zhang, Jeong W. Pak, Shelby Storm, David Alvaro Lopez, Barbara A. Blodi, Amitha Domalpally

**Affiliations:** 1 Wisconsin Reading Center, Department of Ophthalmology and Visual Sciences, University of Wisconsin, Madison, WI, USA

**Keywords:** geographic atrophy, ellipsoid zone, photoreceptor loss, artificial intelligence

## Abstract

**Purpose:**

Ellipsoid zone (EZ) loss is an emerging end point in geographic atrophy (GA) clinical trials. Standardized ground truth is important to train artificial intelligence models in this biomarker. This study compares segmentation and edge detection methods for quantifying EZ and retinal pigment epithelium (RPE) loss on optical coherence tomography (OCT).

**Methods:**

OCT images from 50 eyes with GA were analyzed using segmentation (OCT Explorer) and edge detection (3D Slicer). With segmentation, the EZ and RPE borders were traced to generate thickness maps. For edge detection, graders marked regions of complete EZ or RPE loss on each B-scan. En face maps were generated from both methods and compared. Longitudinal progression was evaluated in 15 eyes with 1 year of follow-up.

**Results:**

The mean EZ loss area was 11.09 ± 5.40 mm^2^ by segmentation and 9.98 ± 5.01 mm^2^ by edge detection (*P* < 0.001). The mean RPE loss area was 7.66 ± 4.40 mm^2^ and 7.48 ± 4.23 mm^2^, respectively (*P* = 0.53) The EZ/RPE ratio was larger with segmentation (1.59 vs. 1.42; *P* < 0.001). Longitudinally, the mean EZ loss change was 1.71 mm²/year vs. 1.45 mm²/year (*P* = 0.43), and the RPE loss change was 1.74 vs. 1.60 mm²/year (*P* = 0.23).

**Conclusions:**

Segmentation and edge detection showed high agreement for EZ and RPE loss, but systematic differences were observed in EZ measurement. Segmentation yielded larger EZ areas by including attenuation over drusen, while edge detection provided more conservative boundaries restricted to GA only.

**Translational Relevance:**

Measurement method influences ellipsoid zone quantification, with implications for trial design and artificial intelligence ground truth development.

## Introduction

Age-related macular degeneration (AMD) has two vision-threatening forms: neovascular AMD (nAMD) and geographic atrophy (GA). Anti-vascular endothelial growth factor therapy is effective for nAMD, but limited treatment exists for GA. The development of therapies for GA requires biomarkers that reflect both structure and function.^1^^,^^2^ Visual acuity is an U.S. Food and Drug Administration (FDA)–approved end point for nAMD, but because GA progression leads to gradual vision loss, earlier markers are needed to capture disease activity before visual decline occurs.^3^^,^^4^

Structural optical coherence tomography (OCT) biomarkers that correspond with function have gained importance in GA.^5^ One such biomarker is the ellipsoid zone (EZ), a hyper-reflective OCT layer representing photoreceptor mitochondrial integrity.^6^^,^^7^ Disruption of the EZ is linked with reduced retinal sensitivity, photoreceptor dysfunction, and precedes both GA progression and measurable vision loss.^8^^–^^10^ The FDA has recently recognized EZ integrity as a potential end point in GA clinical trials.^11^^,^^12^

Measuring EZ loss remains challenging because the EZ signal can be attenuated or absent because of pathology or artifacts. For example, elevation of the retinal pigment epithelium (RPE) by drusen may obscure the EZ band.^13^^,^^14^ Several methods have been developed to quantify EZ integrity. Segmentation traces retinal layer boundaries, provides thickness measurements, and can quantify both thinning and complete loss. However, segmentation may misclassify attenuation from RPE irregularity, such as drusen, which scatter or disrupt the light from the EZ, mimicking true disruption. With segmentation-based methods for EZ loss, there is no flexibility to exclude such confounded regions or mark areas as ungradable. Slab analysis provides en face intensity maps, but results are affected by scan tilt and RPE irregularity.^15^ Edge detection delineates the border between the intact and the absent EZ, reducing false-positive annotation from attenuation, but measurements are limited to complete EZ loss.^2^^,^^16^ Another method is to assess EZ reflectivity maps with signal intensity as a surrogate for photoreceptor health, although overlying lesions such as pseudodrusen can affect results.^14^^,^^17^^,^^18^

Deep learning models increasingly use these methods to train and validate EZ metrics for clinical trials.^8^^,^^15^^,^^18^^–^^22^ In large clinical trials with high-volume imaging, human review of artificial intelligence (AI) outputs is unavoidable. Simplifying this task is essential to minimize grader fatigue and ensure the consistent evaluation of a subtle and challenging end point such as EZ loss. Standardization is, therefore, critical to ensure comparability across studies and algorithms. This study compares two accessible methods—segmentation and edge detection—for measuring EZ loss, with the goal of defining reproducible ground truth generation. These methods were selected based on availability and current use in our workflow, rather than as representative of all existing techniques.

## Methods

### Study Population

Images were obtained from the Wisconsin Reading Center training database of GA cases. Eligible scans included good quality images (distinguishable EZ and RPE layers) with a confirmed diagnosis of GA due to AMD. Eyes with nAMD or other confounding retinal disease were excluded. GA was required to be fully visible within the 6-mm scan region, either subfoveal or nonsubfoveal, and eyes with peripapillary atrophy were not included. This post hoc analysis was approved by the University of Wisconsin Institutional Review Board.

### Image Acquisition and Segmentation

**Figure 1.**
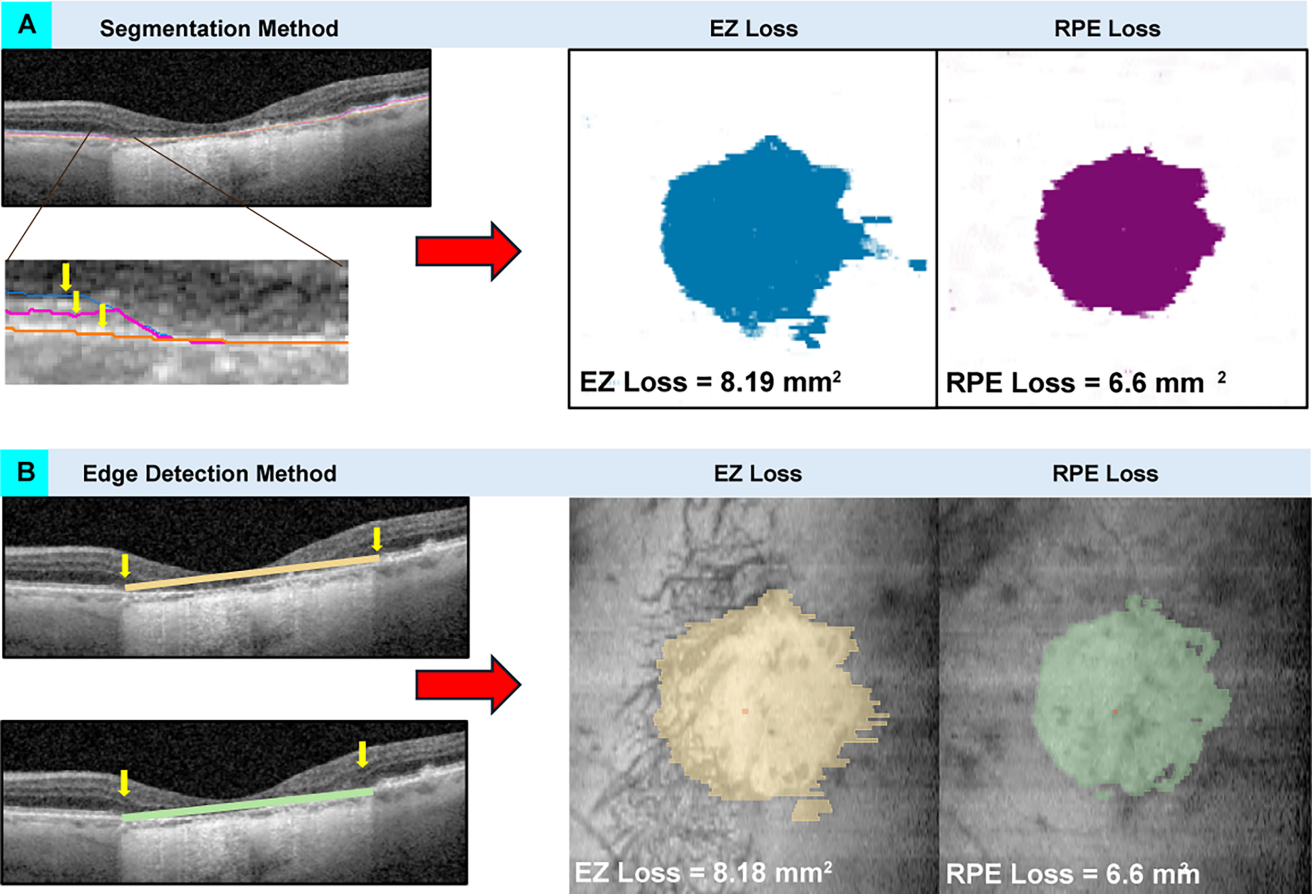
Comparison of segmentation and edge detection methods for EZ and RPE loss. (**A**) Segmentation method. (*Left*) OCT B-scan with layer delineations: *yellow arrows* point to EZ (*blue*), RPE (*pink*), and Bruch's membrane (*orange*). Areas of convergence indicate zones where the EZ and RPE thickness approach zero, typically at GA. (*Right*) En face EZ (*blue*) and RPE thickness (*pink*) maps generated from segmentation across all B-scans. (**B**) Edge detection method. (*Left*) OCT B-scan with *yellow arrows* marking the boundaries of intact vs. absent EZ (*tan bar*) and RPE (*green bar*). (*Right*) En face EZ (*tan*) and RPE loss (*green*) maps generated from annotations across all B-scans.

All scans were acquired using Spectralis SD-OCT (Heidelberg Engineering, Heidelberg, Germany) with a 97 B-scan × 128 A-scan volume protocol. For segmentation, the Iowa Reference Algorithms (Retinal Image Analysis Lab, Iowa Institute for Biomedical Imaging, Iowa City, IA) OCT Explorer 3.8 was used.^23^^–^^25^ The EZ was identified as the second outer hyper-reflective layer between the external limiting membrane and the RPE. Manual segmentation required tracing both the inner boundary of the EZ to the inner boundary of the RPE to calculate EZ thickness, and the inner and outer RPE boundaries to calculate RPE thickness (Fig. 1A).

In areas with drusen or reticular pseudodrusen, segmentation lines followed the visible EZ and RPE contours. For shadowing artifacts, EZ status was inferred from adjacent A-scans. En face thickness maps were generated for EZ and RPE. A threshold of less than 4 µm thickness defined complete loss, so intact EZ appeared white and absent EZ appeared blue on en face maps (Fig. 1A).^19^ This threshold was based on axial resolution of Heidelberg at 3.9 microns/pixel.^26^

### Edge Detection

Edge detection was performed using 3D Slicer 5.6.2 (open-source software).^2^ EZ loss was defined as complete absence of the EZ associated with or adjacent to RPE loss. EZ attenuation over drusen was not considered loss. RPE loss was defined as absence of the RPE band with choroidal hypertransmission.

In Slicer, B-scans and en face views were displayed side by side. A reference plane was established, and each B-scan was inspected for EZ loss associated with GA. Regions with definite EZ loss were annotated with horizontal markers, which were projected to the en face image. The process was repeated across all B-scans to create a two-dimensional map of EZ loss. RPE loss was annotated in the same way. Regions with ambiguous EZ status, such as those with reticular pseudodrusen or drusen, were not part of the assessment (Fig. 1B).

**Table.** Area Measurements and Progression Compared: Segmentation vs. Edge Detection

Features	Edge Detection (mm^2^)	Segmentation (mm^2^)
Mean EZ loss area^*^ (*n* = 50)	9.98 ± 5.01	11.09 ± 5.4
Mean RPE loss area (*n* = 50)	7.48 ± 4.23	7.66 ± 4.4
Mean EZ–RPE^*^ (*n* = 50)	2.50 ± 1.84	3.43 ± 2.27
Mean EZ/RPE^*^ (*n* = 50)	1.42 ± 0.42	1.59 ± 0.51
Mean EZ loss area progression over 1 year (*n* = 15)	1.45 ± 1.12	1.71 ± 2.05
Mean RPE loss area progression over 1 year (*n* = 15)	1.60 ± 1.08	1.74 ± 1.23

* *P* < 0.05.

Values are mean ± standard deviation.

### Dataset and Grading

OCT volumes from 50 eyes were analyzed (20 single-timepoint scans and 15 with both baseline and year 1 follow-up scans). EZ and RPE loss areas were compared between segmentation and edge detection for the 50 eyes. Longitudinal EZ and RPE loss rates were assessed in the 15 paired eyes. Annotations were performed by trained graders (MS, NM, and SZ) at the Wisconsin Reading Center and verified by a senior grader (JPK). Reproducibility was assessed in 10 scans for segmentation and 30 for edge detection.

### Statistical Analysis

All analyses were conducted using R Statistical Software (version 4.5.1; R Core Team, 2025). Areas of EZ and RPE loss were compared between methods using univariable linear mixed models, with a random intercept for the subject to account for correlation between eyes belonging to the same patient. Only one eye per patient was present in the case of available follow-up data, making it appropriate to cluster by subject in all cases. Additional measures included the EZ/RPE loss ratio and EZ–RPE area difference.^20^ Each model was fit with the appropriate area as the outcome variable and the method as a categorical predictor, with segmentation as the reference level. Normality was assessed using Shapiro–Wilk tests in conjunction with quantile–quantile plots. A comparison of growth rates between methods used the Wilcoxon signed-rank test as a nonparametric alternative, where each eye had a single calculated yearly growth rate. Group-level mean values were reported in all cases for consistency. Bland–Altman plots were generated to assess agreement. Statistical significance was defined as a *P* value of less than 0.05. For the linear mixed models, *P* values were calculated using the Satterthwaite approximation for degrees of freedom. Assuming short-term repeatability of approximately 0.5 mm^2^ (approximately 5% coefficient of variation relative to mean EZ loss area), a paired sample size of approximately 34 eyes would be sufficient to detect clinically meaningful differences between the methods.^21^ Spearman's correlation coefficients were calculated to gauge the association between the baseline EZ/RPE ratio and GA growth, as well as between baseline EZ–RPE area difference and GA growth. Intermethod reproducibility was also assessed. The reproducibility of segmentation-based EZ loss has previously been established and was used as a reference standard for comparison with the newer edge detection–based method.^19^^,^^27^

## Results

**Figure 2.**
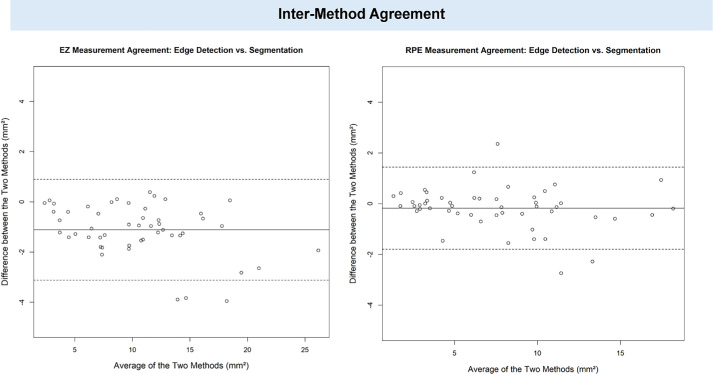
Intermethod agreement. Bland–Altman plots demonstrating the level of agreement between edge detection and segmentation for EZ area measurements (*left*) and RPE area measurements (*right*).

The mean EZ loss area across 50 eyes was 9.98 ± 5.01 mm^2^ by edge detection and 11.09 ± 5.40 mm^2^ by segmentation, with a mean difference of –1.11 mm² (95% confidence interval [CI], –1.72 to –0.50; intraclass correlation coefficient [ICC], 0.96; *P* < 0.001). The mean RPE loss area was 7.48 ± 4.23 mm^2^ and 7.66 ± 4.40 mm^2^, respectively, with a mean difference of –0.17 mm² (95% CI, –0.73 to 0.38; ICC, 0.98, *P* = 0.53; Table 1). Bland–Altman plots (Fig. 2) showed close agreement between the methods for smaller EZ loss areas, with increasing dispersion and larger discrepancies at greater lesion sizes. RPE loss was highly concordant between methods across the measurement range.

The mean EZ/RPE ratio was 1.42 by edge detection and 1.59 by segmentation (*P* < 0.001). The mean EZ–RPE differences were 2.50 mm² and 3.43 mm², respectively (*P* < 0.001). Of the 50 eyes, 15 had 1 year of follow-up. The mean growth rate of EZ loss was 1.45 ± 1.12 mm²/year by edge detection and 1.71 ± 2.05 mm²/year by segmentation (*P* = 0.43). The mean growth rate of RPE loss was 1.60 ± 1.08 mm²/year and 1.74 ± 1.23 mm²/year, respectively (*P* = 0.23).

Supplementary Figure S1 shows the associations between EZ–RPE difference and GA growth, and between EZ/RPE ratio and GA growth, for both the segmentation and edge detection methods on 15 eyes with follow-up. A significant positive association was observed for baseline EZ–RPE difference measured by edge detection (Spearman's *r* = 0.70, *P* = 0.01). Other associations (segmentation-based EZ–RPE difference, and EZ/RPE ratio by both methods) were weaker and not statistically significant (*r* = 0.23–0.43; all *P* > 0.10).

**Figure 3.**
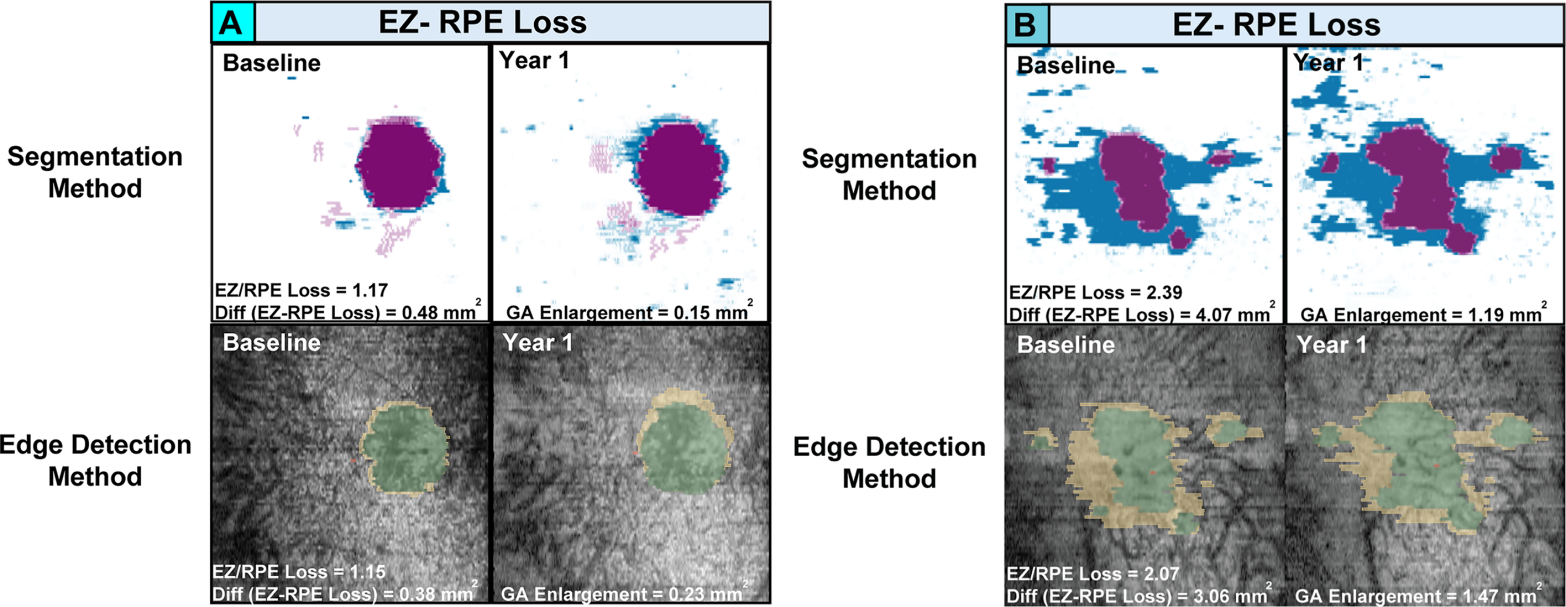
(**A**) Longitudinal comparison between baseline and year 1 with overlay of EZ and RPE loss using segmentation and edge detection methods. Unifocal GA with a small difference between baseline EZ and RPE loss areas at baseline. En face maps show baseline and year 1 with small progression of GA (GA enlargement of 0.15 mm^2^ in the segmentation method and 0.23 mm2 in the edge detection method). (**B**) Longitudinal comparison between baseline and year 1 with overlay of EZ and RPE loss using segmentation and edge detection methods. Multifocal GA with large difference between baseline EZ and RPE loss areas at baseline. En face maps show baseline and year 1 with more progression of GA (GA enlargement of 1.19 mm^2^ with the segmentation method and 1.47 mm^2^ with the edge detection method).

Figure 3A shows an example of an eye with a smaller difference in EZ and RPE loss, and therefore an EZ/RPE close to 1 and a smaller EZ-RPE value, paired with a small amount of GA enlargement at 1 year. Figure 3B shows an eye where EZ loss area is larger than RPE loss, EZ/RPE is greater than 1, EZ-RPE value is larger, and there is greater GA enlargement over 1 year. There was no significant difference in mean EZ loss between the two methods in either unifocal GA (1.2 ± 1.13 mm²; *n* = 18) or multifocal GA (1.1 ± 0.98 mm²; *n* = 32).

### Grader Reproducibility

Intergrader agreement was high for both methods. For edge detection (*n* = 30), the mean difference was 0.10 ± 0.93 mm^2^ for EZ loss and 0.52 ± 0.38 mm^2^ for RPE loss. For segmentation (*n* = 10), the mean difference was 0.61 ± 0.71 mm^2^ and 0.23 ± 0.42 mm^2^, respectively. The ICC exceeded 0.98 for all comparisons.

## Discussion

This study compared two methods of annotating the EZ: segmentation and edge detection. Segmentation is widely used in the literature because there are commercially available software programs to perform segmentation; in contrast, edge detection methods have been previously used to quantify GA area.^16^ Automated methods have also been developed that align conceptually with edge detection. For example, Zhang et al.^28^ trained deep learning models to identify the boundaries of RPE loss, photoreceptor (EZ) degeneration, and hypertransmission, and defined GA as the overlap of these absent regions. Our work extends these approaches by directly comparing manual edge detection of EZ and RPE loss against segmentation in the same dataset, with the goal of clarifying methodological differences and implications for ground truth development for AI training.

**Figure 4.**
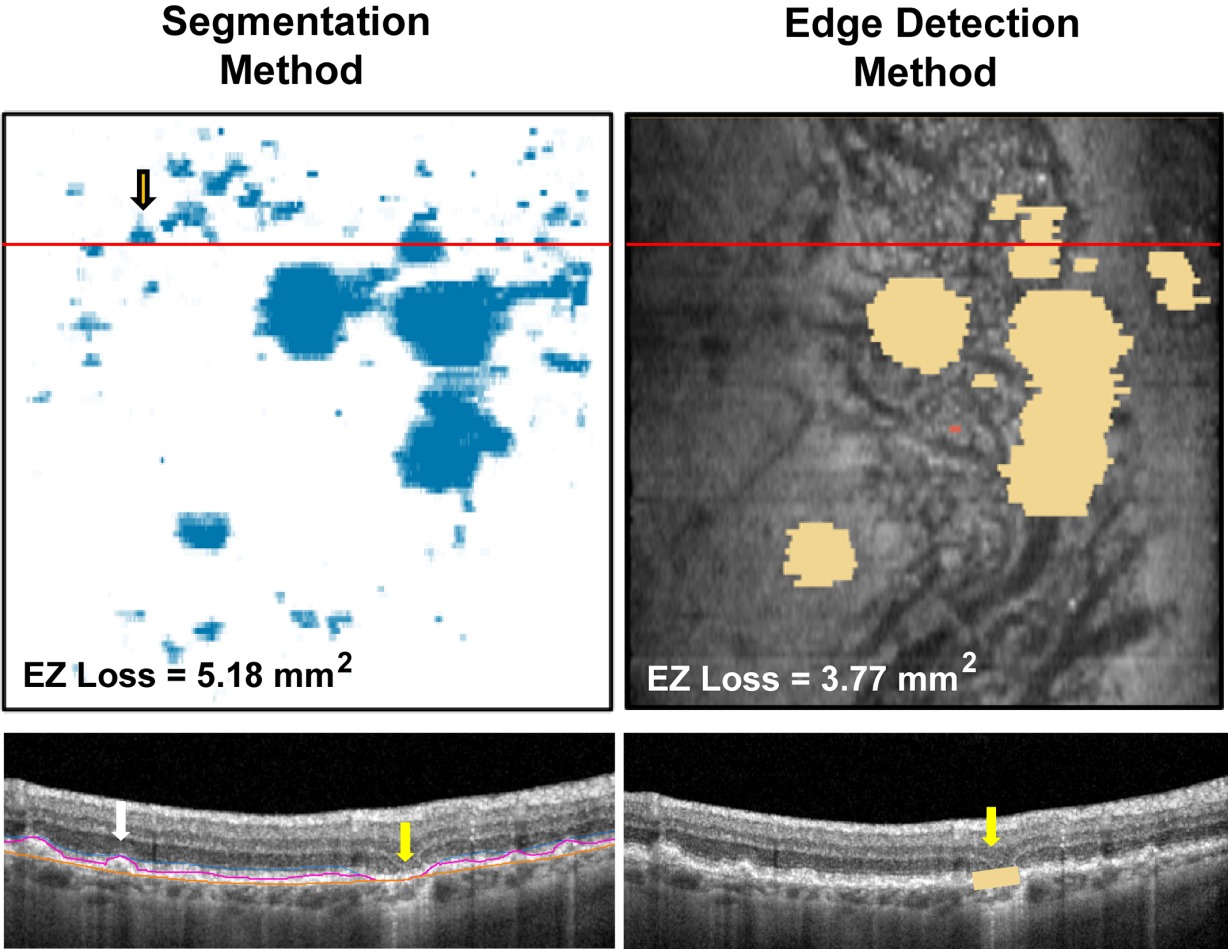
Illustration of differences between segmentation and edge detection in areas of drusen. The en face map of EZ loss derived from segmentation (*blue*) and edge detection (*tan*) is shown. The *red line* corresponds with the B-scan shown below. The *white arrow* indicates EZ loss overlying drusen, where attenuation may be due to true disruption or artifact. The *yellow arrows* indicate true EZ loss in an area without drusen.

Although the available sample size of 50 patients is limited, the data provide key points of discussion for further study. Overall, edge detection produced smaller EZ loss areas than segmentation. This difference reflects methodological distinctions. Edge detection delineates only the transition between intact and absent EZ, whereas segmentation traces the full contour of the EZ band, including regions of RPE irregularity, as shown in Figure 4. Drusen, reticular pseudodrusen, and other RPE irregularities can attenuate the EZ signal and are classified as loss by segmentation. Edge detection avoids this issue by restricting annotation to definite absence of the EZ band, leading to undercalling of EZ loss.

**Figure 5.**
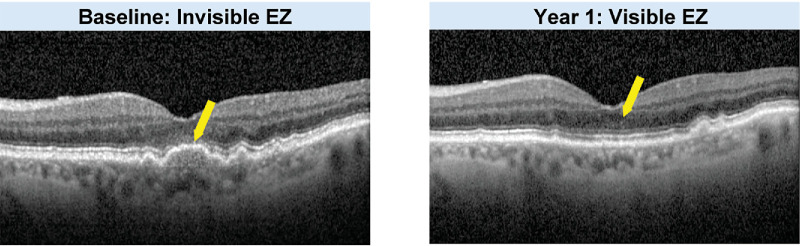
OCT B-scans from the same subject at baseline (*left*) and year 1 (*right*). At baseline, the EZ is not clearly visible beneath overlying drusen (*yellow arrow*). At year 1, the EZ band is clearly visible in the same region (*yellow arrow*), highlighting that EZ signal loss can sometimes reflect attenuation rather than permanent disruption.

These differences have important implications for the use of EZ loss as a clinical trial end point. Drusen are known to be dynamic, with both regression and enlargement occurring naturally over time. As a result, fluctuations in EZ visibility associated with drusen may not reflect true photoreceptor change. Figure 5 illustrates this phenomenon, showing EZ signal loss at baseline overlying drusen that reappears once the drusen regressed at follow-up. Both apparent improvement and worsening of EZ area can occur independent of treatment, creating the potential for false signals in drug trials. Recognizing and accounting for these artifacts is essential when interpreting EZ loss as an outcome measure.

Apart from RPE irregularities, contour handling also differed between the methods for EZ loss. Edge detection applied a conservative boundary that followed exact loss, whereas segmentation extended the boundary over gradual transitions, resulting in broader en face lesions. Segmentation also tended to merge adjacent lesions, whereas edge detection preserved smaller foci as distinct. In contrast, RPE loss was highly similar between methods (7.48 mm² vs. 7.66 mm²), reflecting the more definitive appearance of RPE absence with associated hypertransmission. Both methods tracked RPE loss comparably in longitudinal scans.

Unlike diseases like macular telangiectasis, where EZ loss areas are well defined, assessment of EZ loss in AMD is more subjective.^29^ Our reproducibility findings highlight these differences. Edge detection required graders to identify only definite EZ loss in a limited area of the OCT volume and, thus, resulted in tighter agreement. Segmentation requires assessment of the entire volume scan outside the area of GA and has greater variability. As expected, this difference was not seen for RPE loss assessment, and segmentation performed comparably because subjectivity was minimized.

Recent studies have shown the importance of EZ integrity as a clinical trial end point. Therapeutic effects on EZ loss have been reported to be more pronounced than on RPE loss, and the relative extent of EZ vs. RPE loss can help to identify patients at risk of faster progression.^30^ These findings support the use of EZ-based and composite EZ/RPE measures as sensitive end points in GA trials. In addition, ReCLAIM (Evaluate Safety, Efficacy & Pharmacokinetics of Elamipretide in Subjects With AMD With Non-central GA) demonstrated measurable effects on EZ loss, and EZ attenuation or loss has now been selected as the regulatory-approved primary end point in the phase III elamipretide clinical development program.^31^ Although our sample size was limited, we observed trends consistent with prior studies, including greater EZ–RPE differences and faster progression of GA (Fig. 3; Supplementary Fig. S1). Although the EZ/RPE difference has been shown to be an important predictor, further research using multivariate models is needed to evaluate its relationship with other established risk factors for GA progression, such as lesion multifocality.

## Conclusions

Segmentation and edge detection provided similar results in small GA with clear boundaries, but differences emerged in lesions. Segmentation tended to measure greater EZ loss due to inclusion of RPE irregularities such as drusen, whereas edge detection applied more conservative boundaries. Despite this difference, both methods yielded comparable GA growth rates and comparable measures of RPE loss. For AI training and clinical trial end points, segmentation offers comprehensive layer information but is labor intensive, whereas edge detection can be more efficient and reproducible by focusing on definite loss. Each method, therefore, provides complementary advantages, and understanding their distinctions is essential for defining reliable ground truth and advancing automated analysis in GA clinical trials.

## Supplementary Material

Supplement 1
