# Supplementary material for: Comparison of Measurement Techniques for Photoreceptor Loss in Geographic Atrophy
Source: Transl Vis Sci Technol. 2026 Jul 8;15(7):8. doi: 10.1167/tvst.15.7.8 (PMC13355386; doi:10.1167/tvst.15.7.8)

Supplement 1

Segmentation Method: Scatter plot comparing EZ-RPE difference (top) and EZ/RPE ratio (bottom) to GA growth over 1 year in 15 eyes with longitudinal follow up.


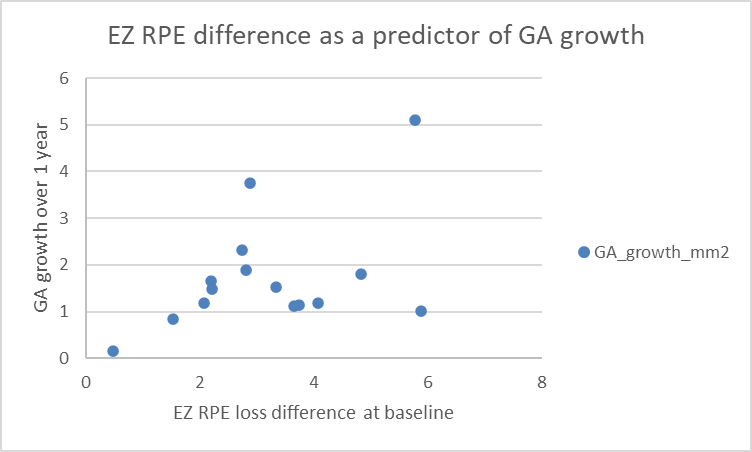

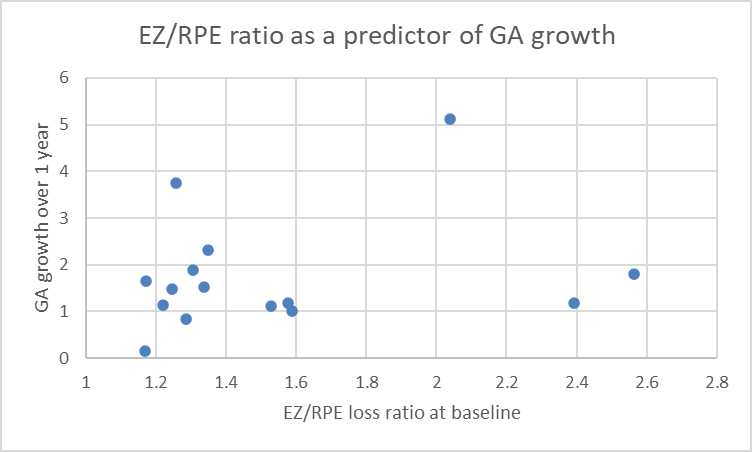


Edge Detection Method: Scatter plot comparing EZ-RPE difference (top) and EZ/RPE ratio (bottom) to GA growth over 1 year in 15 eyes with longitudinal follow up.


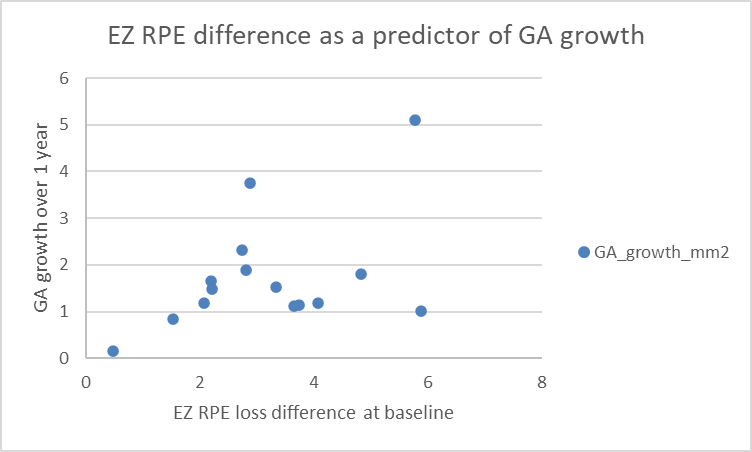

Supplement: Supplement 1 [file tvst-15-7-8_s001.docx]
